# Canopy Height and Epiphytic Bryophytes Shape Fungal Communities in a Temperate Rainforest

**DOI:** 10.1002/ece3.72241

**Published:** 2025-10-02

**Authors:** Laurel Renee Humphreys, Jane M. Lucas, Michelle Elise Spicer

**Affiliations:** ^1^ Department of Ecology & Evolutionary Biology Yale University New Haven Connecticut USA; ^2^ Department of Earth & Environmental Sciences Lehigh University Bethlehem Pennsylvania USA; ^3^ Cary Institute of Ecosystem Studies Millbrook New York USA; ^4^ Yale School of the Environment New Haven Connecticut USA

**Keywords:** biotic interactions, host specificity, microbiome, nonvascular epiphyte, plant–microbe interactions, vertical gradient

## Abstract

Fungal communities contribute to plant ecology and evolution in forested ecosystems. Their diverse interactions with associated host plants can vary along abiotic and biotic gradients, but these gradients are poorly understood in complex natural ecosystems. Given the high diversity of epiphytic plants in many ecosystems, forest canopies offer a unique and underexplored system for studying plant‐associated microbial diversity and distribution. We explored both abiotic and biotic factors structuring arboreal fungal communities. Specifically, we hypothesized that bryophyte‐associated fungal communities are structured by the vertical height gradient within host trees (from the ground to high in the canopy), vary across host plant species, and that living bryophytes host distinct fungal communities compared to dead bryophyte matter. To test these hypotheses, we sampled living and dead bryophytes (mosses and liverworts) across three different bryophyte host species and four heights, ranging from the forest floor to 18 m above the ground. We characterized the fungal community composition in each sample using metabarcoding. Fungal communities showed significant variation across substrates: bryophytes collected from the ground exhibited 17% greater Shannon diversity and 34% higher taxonomic richness than epiphytic bryophytes, while living bryophytes had 15% higher diversity and 30% greater richness than dead tissues. This pattern suggests that the diverse microhabitats within living bryophytes may drive microbial diversity. Community analysis identified a core fungal community across living bryophyte samples, but rare taxa accounted for a majority of reads, driving differences in community composition between different heights and bryophyte species. Synthesis: Epiphytic bryophyte‐associated fungal communities show high heterogeneity across different substrates and heights, which provides insight into the structuring of the forest microbiome and epiphyte ecology. These results demonstrate the importance of exploring canopy‐associated microbes to better understand microbial diversity and function in forest ecosystems.

## Graphical abstract

We present the first investigation into epiphytic bryophyte‐associated microbes in a temperate ecosystem. Our field study reveals taxonomic and functional differences between ground‐ and canopy‐associated fungi. Host plant specificity and bryophyte senescence drive fungal diversity and community composition, indicating strong biotic drivers for fungal distribution.

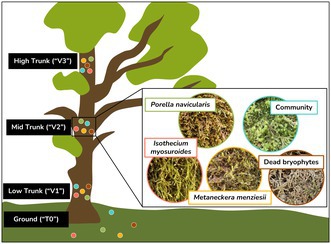

## 1 Introduction

Forest canopies host a remarkable diversity of fungi, representing a mosaic of biotic and abiotic conditions that enable fine‐scale investigations into fungal communities (Harrison et al. 2016). These canopy‐associated fungi interact with plants in complex ways, influencing nutrient acquisition, stress tolerance, growth, and susceptibility to pathogens (Sharma et al. 2020; Trivedi et al. 2020). By mediating such processes, fungal communities are key regulators of plant diversity, productivity, and distribution, potentially shaping large‐scale patterns of plant diversity (Maron et al. 2011; Pellissier et al. 2013; Shinohara et al. 2024; Wang et al. 2019, 2022). However, despite their ecological importance, the major drivers of fungal community composition and diversity within forest canopies remain poorly understood.

Both fungi and epiphytic plants in the canopy are subject to unique abiotic stressors compared to the ground. Tree canopies can be harsh environments, with higher sunlight exposure (Gora et al. 2019; Looby et al. 2020), more dramatic fluctuations in moisture and temperature (Aubrey et al. 2013), and limited nutrients (Hietz et al. 2002) compared to the ground. Fungi are sensitive to changes in moisture and temperature (Allison and Treseder 2008) and are likely shaped by this gradient. Ground substrates provide high mineral content, whereas canopy substrates are composed of organic materials such as tree bark, decaying plant matter, and living organisms, with low availability of inorganic nutrients (Ingram and Nadkarni 1993; Benzing 1990). Even within a single tree, the microenvironment can vary significantly from the ground to the upper canopy, and from the main trunk to the outer branches (Woods et al. 2015, 2019; Spicer and Woods 2022). In addition, the growth of fungal hyphae in the canopy is restricted to the available space on the trunk, branch, and epiphytic plants, and separated by meters of open air, whereas fungi growing along the continuous solid of the forest floor can grow almost indefinitely. Fungal assembly in the canopy may thus be limited by environmental filtering, through phenomena such as the disinfecting effects of sunlight in the canopy (Parker et al. 1995) and dispersal limitation at greater heights (Gilbert 2005). Height in the canopy could be a determining factor of fungal assembly, but the direct effects of abiotic variation along this vertical gradient have scarcely been explored.

In addition to the aforementioned abiotic controls in canopy ecosystems, microbial diversity and structure may also be impacted by biotic factors such as host plant availability (Davey et al. 2013; Cook et al. 2022) and host plant specificity (Li et al. 2020; Apigo and Oono 2022). The structural heterogeneity of large trees, in addition to the microclimate shifts observed in the canopy, enables many different epiphytic plant species to coexist (Woods et al. 2019). Certain bryophyte hosts are more abundant at different positions within the canopy (Woods et al. 2019), potentially influencing the distribution of epiphytic plant‐associated fungi. Many fungi seem to display some degree of specialization to one or more plant host species (Davey et al. 2013), which could indicate that certain hosts are more readily colonized and/or nutritionally rich than others. On the other hand, certain fungi appear to be host generalists whose distribution and high spatial heterogeneity are impacted more strongly by stochastic processes than host plant traits (Cook and Taylor 2023). Though high host specificity has been observed in ground‐rooted ecosystems (Davey et al. 2013; Kembel and Mueller 2014), there are few studies examining patterns of fungal host specificity in the canopy (Cook and Taylor 2023).

Even within one host plant species, microbial community composition, biomass, and species richness can vary between tissue types. Different parts of a single plant possess unique tissues, and thus distinct fungal habitats. Many bryosymbiotic ascomycetes show high niche selection, occupying distinct and species‐specific microsites on their bryophyte hosts (Döbbeler 2002). Some fungal taxa in Basidiomycota seem to be more abundant and active in senescing and dying tissues than in photosynthetic tissues in bryophytes on the forest floor (Davey et al. 2013; Chen et al. 2018). In the one study that directly tested fungal communities in living and dead bryophytes in tropical canopies, living bryophytes harbored higher fungal diversity than dead bryophytes (Cook et al. 2022). Living bryophytes could provide opportunities for fungal niche differentiation through substrate and nutrient preferences. To our knowledge, no studies have investigated fungal communities among living and dead epiphytes in temperate canopies, despite the unique opportunities provided by these varying tissue types on a small spatial scale.

Fine‐scale differences in fungal communities across height, host plant species, and host tissue type, along with abiotic conditions, may drive the function, growth, and distribution of canopy plants. Canopy systems are often overlooked in plant–microbe interactions, despite their unique plant communities and microenvironmental pressures. In this study, we address these knowledge gaps by exploring the combined effects of canopy height, host plant species, and plant tissue type on epiphytic bryophyte‐associated fungal communities in temperate rainforest canopies. To accomplish this, we sampled living bryophytes and senescent bryophyte tissue across different bryophyte species and heights from the ground, including on the forest floor, and characterized the fungal constituents in each sample using metabarcoding. We had three hypotheses. First, we hypothesized that the abiotic variation from the forest floor to the canopy drives fungal community composition, abundance, and diversity. We predicted that fungal diversity would decrease higher in the canopy due to the high sunlight and aridity and lack of access to ground soils. Second, we examined whether canopy fungi show high host specificity, predicting that bryophyte species will affect fungal community composition. Third, we tested whether living and senesced bryophyte tissues host functionally and taxonomically distinct fungal communities. We predicted that living bryophyte tissue would have higher fungal diversity and higher community dispersion than dead bryophytes due to the greater amount of distinct fungal niches in active tissue. We also expected to find differences in primary lifestyle among fungi associated with different heights, plant hosts, and tissues. For example, dead plant tissue could host more saprotrophic fungi, while living tissues may be more likely to host endophytes or parasites. This study provides insight into the biotic and abiotic drivers of fungal community assembly in the canopy, which is crucial given the impact of fungi on host plant success and distribution.

## 2 Materials and Methods

### 2.1 Study Site

All field work was performed in the Olympic Peninsula's Hoh Rainforest, in Olympic National Park, WA (47.8569° N, 123.9215° W). This site is in a temperate rainforest dominated by nonvascular epiphyte‐laden old‐growth trees, in a region that has been previously studied for epiphyte nutrient cycling and vertical gradients in epiphyte communities (e.g., Woods et al. 2019; Rousk and Nadkarni 2009). Climbing infrastructure was already in place for long‐term canopy research in 12 adult bigleaf maple trees ( *Acer macrophyllum* ) in an old riparian zone near the Hall of Mosses (funded by NSF‐PRFB #1907190 to M.E.S.). Heavily epiphyte‐laden trees were selected that were safe to climb using a modified single‐rope climbing method (Perry 1978).

### 2.2 Microbial Community Sampling

**FIGURE 1:**
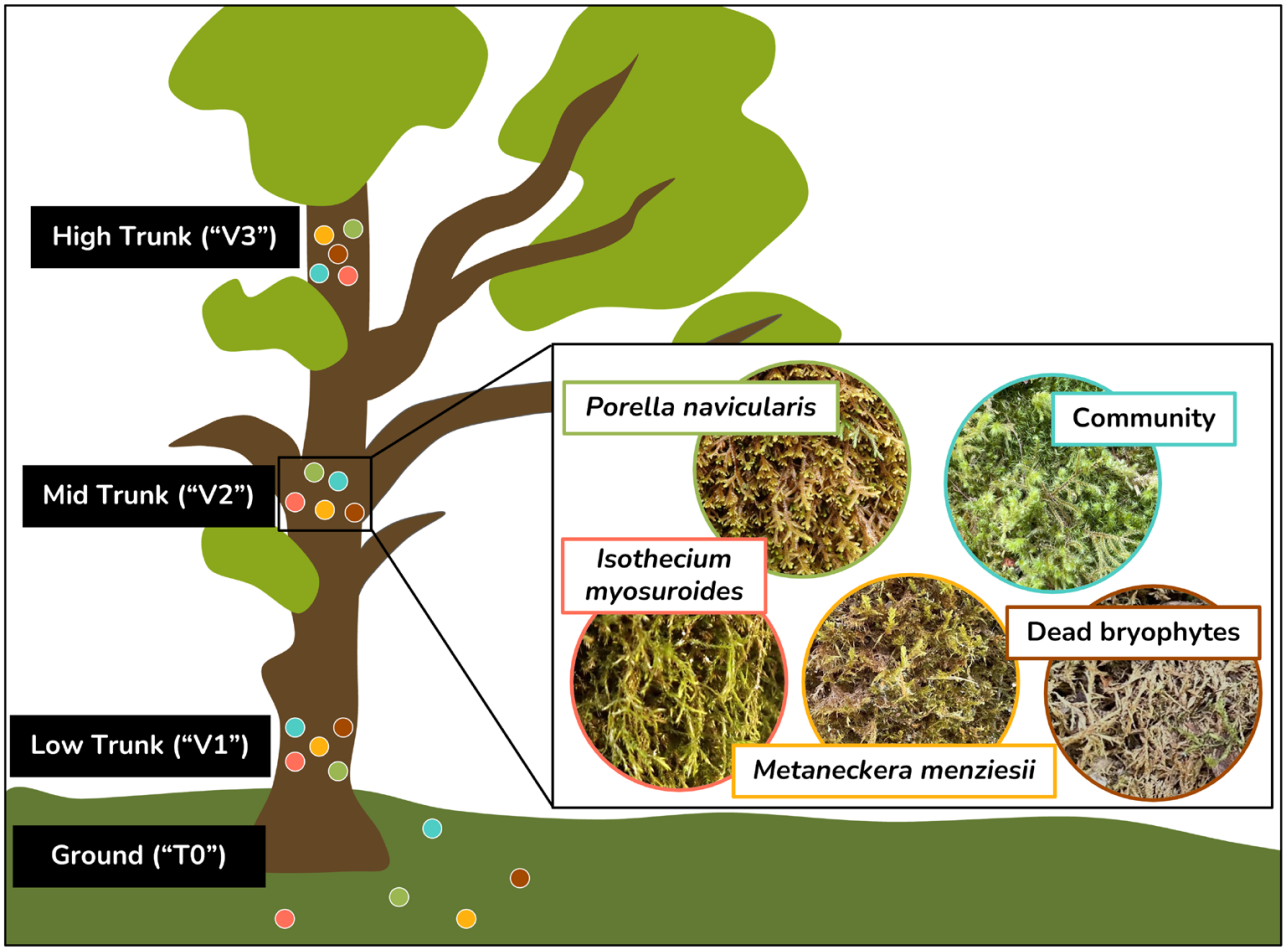
Sampling design demonstrating the vertical structure of bryophyte collection and samples taken from each height zone: *Isothecium myosuroides* , *Metaneckera menziesii* , *Porella navicularis* , a pooled community sample, and dead bryophyte matter.

To examine the diversity and distribution of fungal communities associated with epiphytic bryophytes, we sampled bryophytes across a range of substrate types in a fully factorial design that included different vertical positions in the canopy, bryophyte species, and living and dead bryophytes. We collected bryophyte samples from the ground and three canopy heights at each of 12 *A. macrophyllum* trees (Figure 1, Table A1). Five ~150 mg samples were taken at each location, including three focal bryophyte species, one pooled community sample, and one sample of dead bryophyte tissue.

To characterize the variation across heights, we sampled bryophytes in plots at three different height zones on the trunk (Johansson Zones “JZs 1–3”; Johansson 1974), as well as from a reference plot on the ground “T0” (i.e., 4 vertical positions in total). Sampling plots in the tree were randomly selected in a blocked design; the lowest height zone ranged from the ground to 2 m (JZ1; “V1”; 0.5–1.9 m), the second from 2 m to the first bifurcation of the branches (JZ2; “V2”; 3.8–7.2 m), and the highest from the first branch to the highest accessible area within the tree crown (JZ3; “V3”; 9.4–17.8 m).

To characterize fungal variation among living bryophyte substrates, we collected three living focal bryophyte species ( *Isothecium myosuroides* , *Metaneckera menziesii* , *Porella navicularis* ) and a pooled community sample of nearby live nonfocal bryophyte species. The three focal species were previously found to be among the most widespread and abundant bryophyte species at various height zones, based on surveys of the focal trees (Spicer, unpublished data) and at a nearby field site (Woods et al. 2019). We selected these species because they were all likely to be present in or near each of our plots and had distinct substrate preferences and natural histories. We included the community sample to characterize the fungal communities associated with less common species. Each community sample included three to six nonfocal bryophyte species found within or near the plot, allowing us to control for species‐specific factors that might influence microbial communities. We documented the species included in each community sample. There were over 20 bryophyte species represented in our community samples, and the bryophytes present within each sample varied strongly between sites. To test for differences between living and dead bryophytes, we also collected dead bryophyte (“necromass”) samples from beneath living bryophyte mats. Living bryophytes were identified to species using *Plants of the Pacific Northwest Coast* (Pojar and MacKinnon 2014) and/or *Mosses, Lichens & Ferns of Northwest North America* (Vitt et al. 1988). The few unknown bryophytes in the community samples were identified to genus and assigned a standardized morphotype name.

We placed samples into ZR Bashing Bead Lysis Tubes (Zymo Research Inc.) using 70% ethanol‐sterilized forceps. The lysis tubes were pre‐filled with DNA/RNA shield (Zymo Research Inc.) to immediately stabilize microbial DNA in situ and minimize community turnover during transport. We agitated each sample at 3800 rpm for 30 s using a Wig‐L‐Bug grinding mill (Sigma‐Aldrich; Z529648) to pulverize microbial cells. Due to the varying availability of certain species at each plot and the challenges of microbial sampling while climbing, there were minor differences in the amount of bryophyte mass in each sample tube. Samples were normalized during sequencing preparation and data processing, as described below.

### 2.3 Molecular Methods

We extracted fungal DNA using a Zymo Fecal/Soil Microbe Miniprep Kit (Zymo Research Inc.) according to the manufacturer's protocol beginning at Step 3. We amplified the fungal ITS1 and 2 regions in accordance with the Earth Microbiome Project's protocol (Smith et al. 2018). Although ITS primers have known biases, particularly for mycorrhizal taxa, we selected them to maximize overall fungal diversity. The amplification conditions were as follows: an initial denaturation at 94°C for 1 min, 35 cycles of 94°C for 30 s, 52°C for 30 s, and 68°C for 30 s, followed by a final step of 68°C for 10 min. Unique DNA barcodes were attached in a subsequent PCR reaction to allow for later pooling. Barcoded amplicons were cleaned and normalized using SequalPrep Normalization Plates (ThermoFisher Inc., Massachusetts, USA; Harris et al. 2010). Amplicons were pooled together, then sequenced using an Illumina MiSeq instrument with 600 v3 chemistry. Multiple blank samples were processed alongside field samples as a control.

### 2.4 Bioinformatics

Sequencing of 238 samples produced 4,938,857 raw ITS gene reads (2 samples were dropped due to sequencing errors). Raw ITS gene reads were quality filtered and assigned to Amplicon Sequencing Variants (ASVs) using the DADA2 pipeline (Callahan et al. 2016). Taxonomy was assigned using the UNITE database, version 9.0 (Nilsson et al. 2019; Abarenkov et al. 2022). ASVs unassigned at the phyla level or identified as contaminants using the decontam package in R were removed (Davis et al. 2018). We then rarefied samples to 2072 sequences per sample to control for uneven sequencing depths. 42 samples were dropped due to read counts that were below this threshold. Our final sample number for ITS gene analyses was 196 samples, comprising 406,112 reads (Table A2). We assigned functional guilds to ASVs in the rarefied dataset using the FungalTraits database, which matched 50% of ASVs to a primary lifestyle by genus (Põlme et al. 2020; Tanunchai et al. 2023). Half of the fungal taxa in our samples were not represented in the FungalTraits database. This is consistent with the rate of functional assignment in other publications relying on fungal functional databases (Tanunchai et al. 2023).

### 2.5 Statistical Analysis

Analyses were performed using the R statistical environment, version 4.4.0 (R Core Team 2024). Microbial sequencing data manipulation and analysis were performed using the packages *phyloseq* (Leff 2022), *mctoolsr* (McMurdie and Holmes 2013), and *microbiome* (Lahti and Shetty 2024). We examined microbial community structure using the packages *vegan* (Oksanen et al. 2024) and *microbiome* (Lahti and Shetty 2024). Linear mixed effects models were carried out using the *lme4* and *lmerTest* packages (Bates et al. 2015; Kuznetsova et al. 2017). We assessed fit by using residual QQplots (*DHARMa* package, Hartig 2022) and comparing AIC between models. Pairwise post hoc comparisons for linear mixed effects models were performed using the *emmeans* package (Lenth 2024).

To test alpha diversity differences between heights and bryophyte species, we used a subset of our data that only included living bryophyte samples. Dead bryophyte samples were excluded because they included multiple undetermined species of bryophytes. We incorporated categorical height (T0/V1/V2/V3), species ( *Isothecium myosuroides* “ISOMYO,” *Metaneckera menziesii* “METMEN,” *Porella navicularis* “PORNAV,” and Community “COM”), and their two‐way interactions as categorical fixed effects. The unique tree identifier (TreeID) was included as a random effect to account for the nested structure of the height categories, improving fit for all models. There were no significant interactions between fixed effects, so interactions were dropped from the final models. We determined variation between heights and species by obtaining estimated marginal means and applying a Tukey adjustment for pairwise comparison of means. To evaluate diversity differences between living and dead bryophytes, we analyzed the full dataset and used status (Alive/Dead) and categorical height as fixed effects, with TreeID as a random effect. Once again, interactions were not significant and were dropped from the final models. We used mixed effects models of this construction to compare ASV richness, Chao1 richness (Chao 1984), and Shannon–Weiner diversity (Shannon and Weaver 1949). Results were determined to be significant when *p* < 0.05.

We transformed microbial total sequencing reads into relative abundances before calculating Bray–Curtis dissimilarity. We then used these community distance matrices to carry out NMDS (nonmetric multidimensional scaling) ordinations. We used a subset of only living bryophyte samples to assess the impacts of height and bryophyte species on fungal communities, and we included all samples in our assessment of bryophyte status. We performed PERMANOVA analysis using the *adonis2* function (package *vegan*) to assess the impact of height, host species, and bryophyte status on fungal community composition. We used post hoc pairwise PERMANOVAs to determine which pairs had significant differences (package *pairwiseAdonis*; Martinez Arbizu 2017), using a Bonferroni correction to correct for multiplicity. We compared beta diversity between heights, bryophyte species, and bryophyte status using PERMDISP beta dispersion tests (package *vegan*) for the analysis of multivariate homogeneity of group dispersions.

To test for differences in the relative abundance of fungal functional groups between samples, we used linear models of a similar construction as described above. We calculated the relative abundance of each primary lifestyle (e.g., wood saprotroph, plant pathogen, lichenized, etc.) within each sample. To meet the assumptions of our statistical models and facilitate interpretation, we logit‐transformed the relative abundance data for each fungal functional group. To compare the logit‐transformed relative abundance of each fungal functional group across height and bryophyte species, we incorporated height and bryophyte species as fixed effects and TreeID as a random effect. To compare bryophyte status, bryophyte status was included as a fixed effect, with TreeID as a random effect. We compared fungal taxonomic changes using the same construction, with the logit‐transformed relative abundance of each of the 10 most abundant fungal orders as the dependent variable. We also tested for significant associations between specific fungal orders or functional groups with corresponding heights, bryophyte species, and status with indicator species analyses (package *indicspecies*, De Cáceres and Legendre 2009).

## 3 Results

### 3.1 Fungal Community Composition and Function Across a Vertical Gradient

**FIGURE 2:**
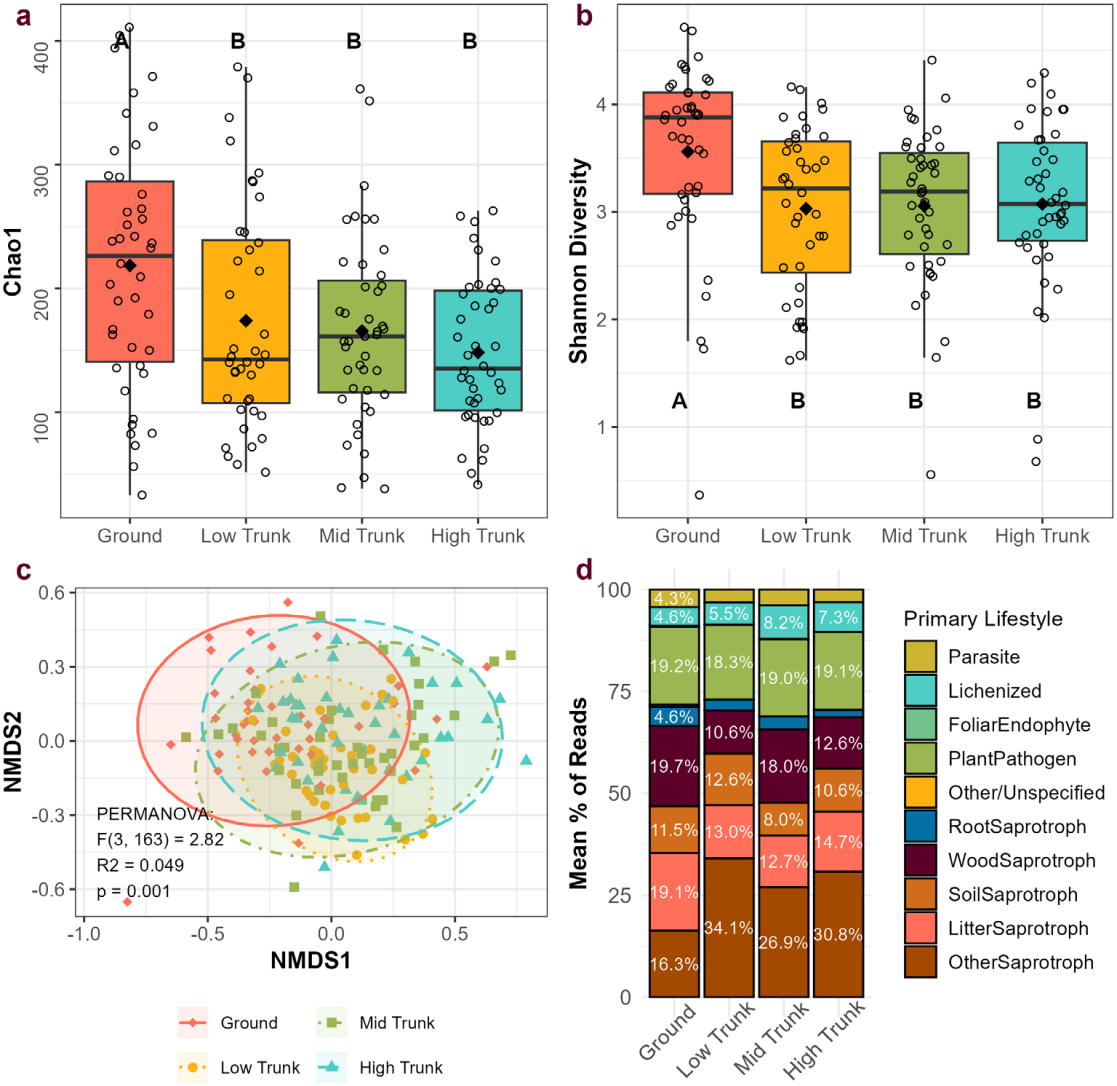
Diversity, community composition, and function in living bryophyte samples across heights. (a) Boxplot showing Chao1 richness across height. A/B/B/B indicates a significant difference between ground‐level samples and all epiphytic bryophyte samples (*F* (3, 150.2) = 6.2, *p* < 0.001). (b) Boxplot showing significant differences in Shannon diversity between ground and tree samples (*F* (3, 150.4) = 4.51, *p* = 0.005). (c) Ordination of fungal community structure grouped by height (stress = 0.17), with PERMANOVA results (calculated using Bray–Curtis dissimilarity). Ellipses represent 95% confidence intervals for each group. (NMDS 1 and 3 in Figure A1). (d) Stacked bar chart demonstrating the average percentage of each functional group in samples of each height.

Fungal communities differed across the vertical gradient, with ground samples often exhibiting the highest levels of diversity. Specifically, ground‐level samples had higher taxonomic richness, Chao1 richness, and Shannon diversity than samples taken from anywhere on the trunk (Figure 2a,b, Table A3). Pairwise comparisons indicated that within‐tree samples were similar in alpha diversity (*p* > 0.33). Ground‐level samples had, on average, 34% higher richness, 35% higher Chao1, and 17% higher Shannon diversity than samples of epiphytic bryophytes (Table A3).

**FIGURE A1:**
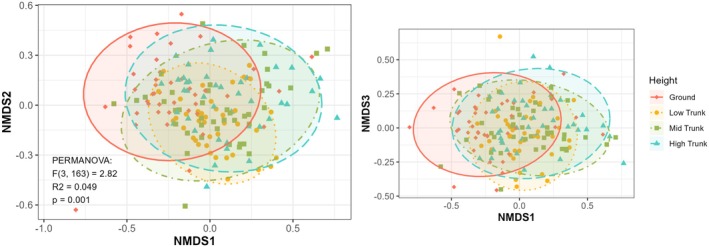
Ordination of fungal community structure grouped by height (stress = 0.17). Ellipses represent the 95% confidence region for the group.

Consistent with our predictions, community composition also varied across the vertical gradient (Figures 2c, A1, A4 and Table A4). Pairwise comparisons within the canopy showed differences in community composition between the low trunk and high trunk (pseudo‐F = 3.22, *R* ^2^ = 0.04, *p* = 0.001), and between the ground and all canopy levels (pseudo‐F > 3.37, *R* ^2^ = 0.04, *p* = 0.001). This compositional change was reflected in the differences in abundance of certain fungal orders between heights (Figure A4, Table A5). For example, the fungal order Cystofilobasidiales was 18% less abundant in the highest levels of the canopy compared to ground‐level and low trunk samples (*t* > 2.7, *p* < 0.037), while Capnodiales and Pleosporales were 2 and 2.5 times as abundant, respectively, on the high trunk compared to the ground (*t* > 2.8, *p* < 0.027) (Table A5). Indicator species analyses identified 59 fungal genera associated with ground‐level samples, 14 with low trunk samples, 5 with mid trunk samples, and 7 with high trunk samples (Table A12). The high number of significant associations with ground‐level samples is consistent with the observed pairwise differences in community composition. These taxonomic shifts were accompanied by shifts in the relative abundance of functional groups (Table A6, Figure 2d). Wood saprotrophs were 56% more abundant on the ground than in the high canopy (*t* = 2.87, *p* = 0.024), and litter saprotrophs were 50% more abundant on the ground than the mid canopy (*t* = 2.66, *p* = 0.042) (Figure 2d, Table A6). These two functional groups were identified as having significant associations with ground‐level samples in an indicator species analysis (Table A11). A core community of 31 fungal taxa (identified as having over 1% abundance in over 50% of living samples) accounted for only 24% of reads in ground‐level samples, compared to 40% in aboveground samples.

### 3.2 Fungal Community Changes Across Bryophyte Species

**FIGURE 3:**
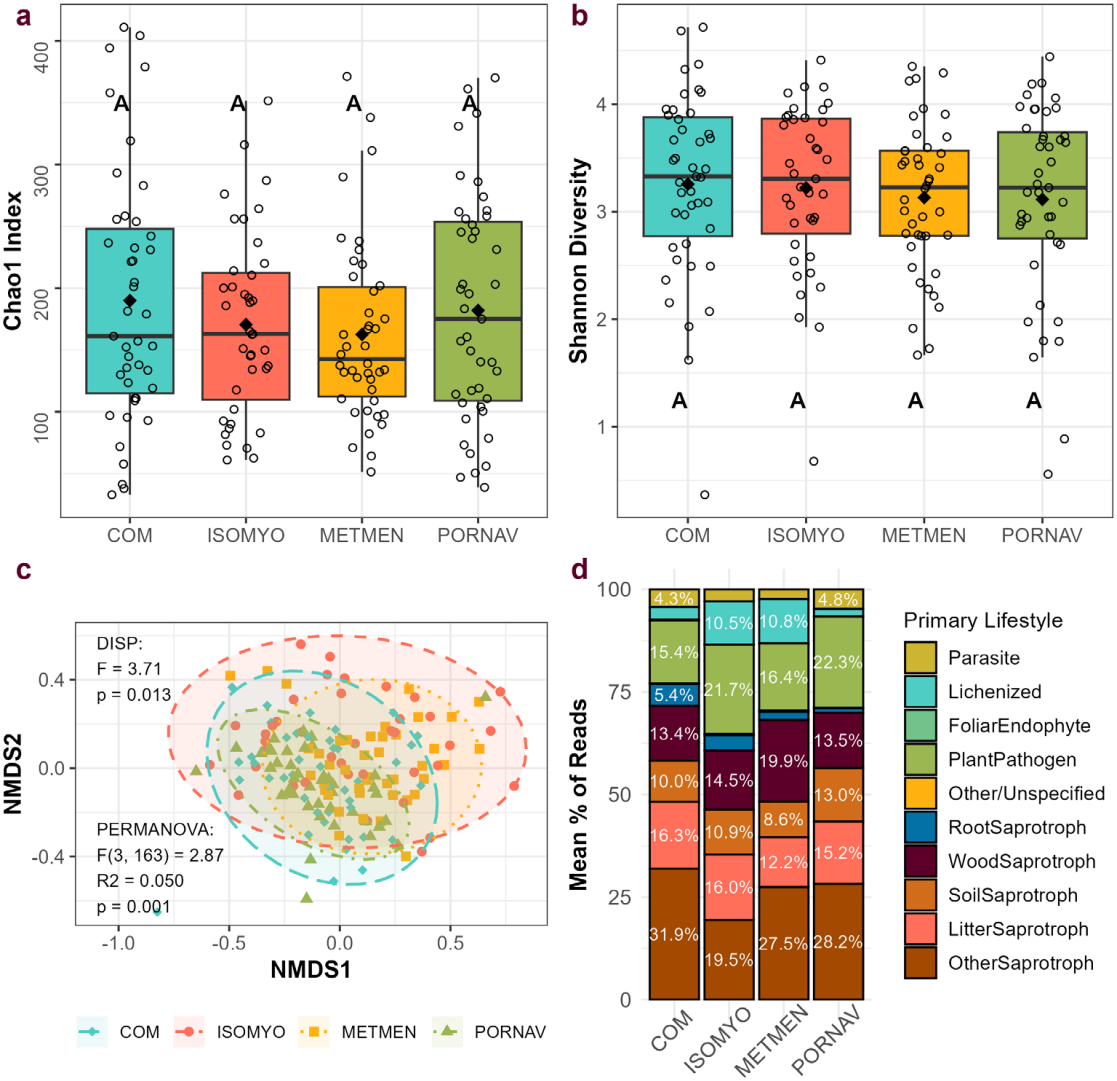
Diversity, community composition, and function across host bryophyte species. (a) Boxplot showing Chao1 richness across species. A/A/A/A indicates lack of significant difference between bryophyte species (*F* (3, 150 = 0.91), *p* = 0.43). (b) Boxplot showing no significant differences in Shannon diversity across bryophyte species (*F* (3, 150 = 0.29), *p* = 0.83). (c) Ordination of fungal community structure grouped by species (stress = 0.17), with PERMANOVA results (calculated using Bray–Curtis dissimilarity) and PERMDISP results indicating differences in community composition and dispersion. Ellipses represent 95% confidence intervals for each group. (NMDS 1 and 3 in Figure A2). (d) Stacked bar chart demonstrating the average percentage of each functional group in each bryophyte species.

**FIGURE A2:**
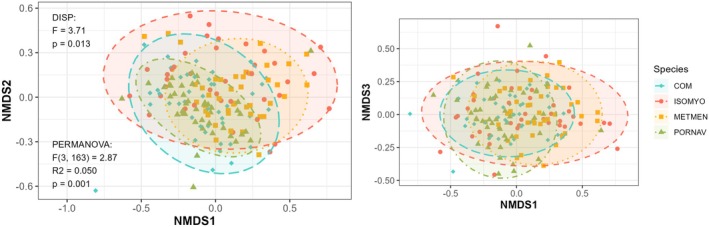
Ordination of fungal community structure grouped by bryophyte species (stress = 0.17). Ellipses represent the 95% confidence region for the group.

Different bryophyte species hosted taxonomically and functionally distinct communities of fungi. Fungal community composition varied by host bryophyte species, and this difference was significant across all pairwise comparisons (pseudo‐F > 2, *R* ^2^ > 0.025, *p* = 0.001, Table A4). The permutational analysis of multivariate dispersion also indicated significant differences in dispersion among species (Figure 3c, Figure A2). Compositional changes were reflected in the relative abundances of different orders of fungi (Figure A4). Fungi in the order Lecanorales, which contains lichen‐forming fungi, were significantly more abundant in *I. myosuroides* and *M. menziesii* samples compared to *P. navicularis* and community samples (*t* > 3.3, *p* < 0.007) (Figure A5 and Tables A7, A11). On average, the relative abundance of lichenized fungi was 4.3 times greater in *I. myosuroides* and *M. menziesii* samples compared to *P. navicularis* and community samples (*p* < 0.001) (Figure A5, Table A8). Lichen parasites, on the other hand, were over 3 times as abundant in *P. navicularis* and community samples than *M. menziesii* samples (*p* < 0.005) (Table A8). Indicator species analyses identified 6 genera associated with *I. myosuroides* , 7 with *M. menziesii*, and 26 with *P. navicularis* (the only liverwort species) (Table A12). Despite these compositional differences, all measures of alpha diversity were similar across bryophyte species (*F* (3, 150) < 0.92, *p* > 0.43) (Figure 3a/b, Table A3).

### 3.3 Effect of Bryophyte Status on Fungal Diversity and Composition

**FIGURE 4:**
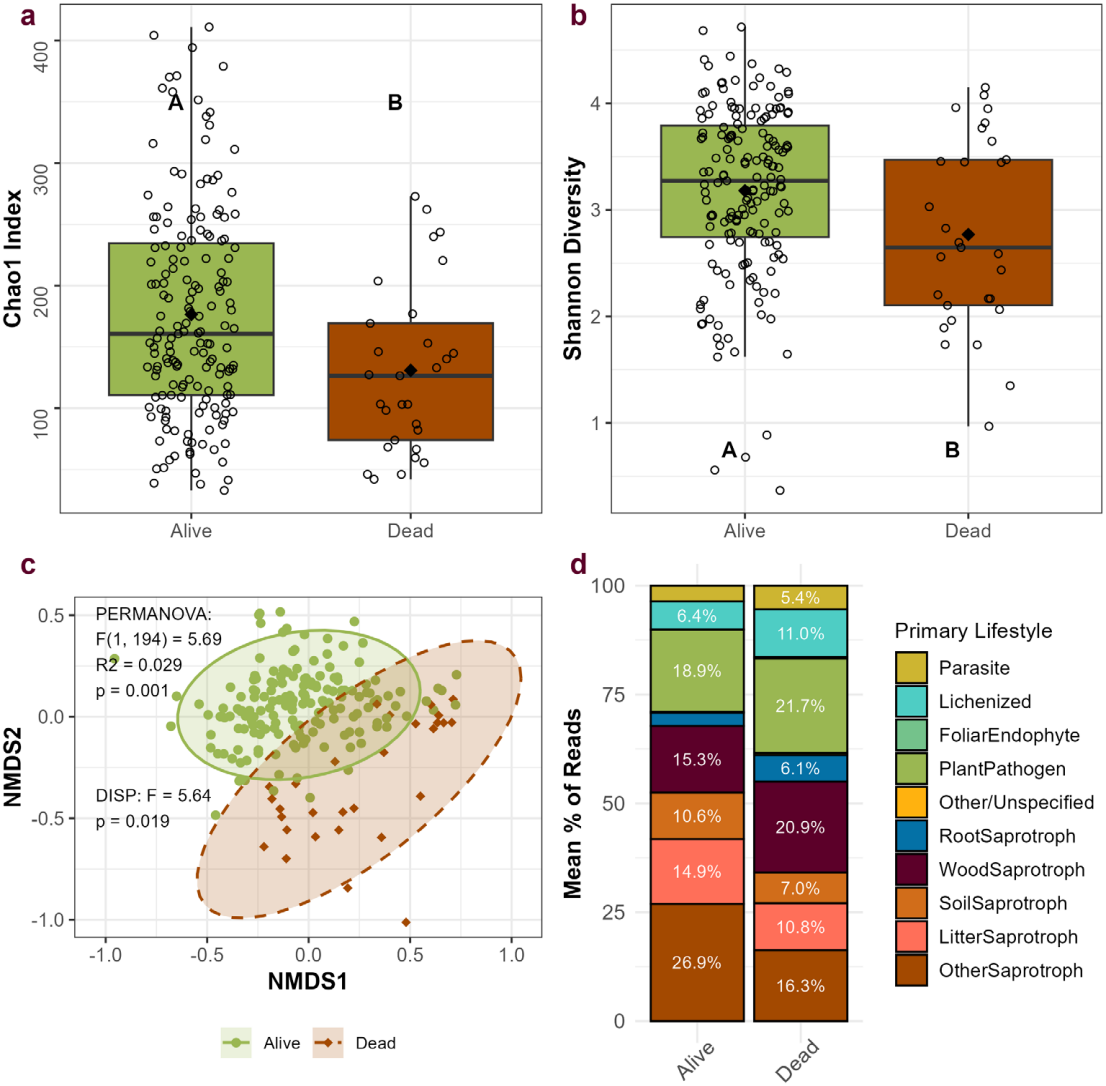
Diversity, community composition, and function across host bryophyte status. (a) Boxplot showing Chao1 richness in living and dead samples. A/B indicates significantly higher Chao1 in living samples (*F* (1, 183) = 10.54, *p* = 0.001). (b) Boxplot showing significantly higher Shannon diversity in living bryophyte samples (*F* (1, 184) = 8.74, *p* = 0.004). (c) Ordination of fungal community structure grouped by species (stress = 0.17), with PERMANOVA results (calculated using Bray–Curtis dissimilarity) and PERMDISP results indicating differences in community composition and dispersion. Ellipses represent 95% confidence intervals for each group. (NMDS 1 and 3 in Figure A3). (d) Stacked bar chart demonstrating the average percentage of each functional group in living and dead bryophyte samples.

**FIGURE A3:**
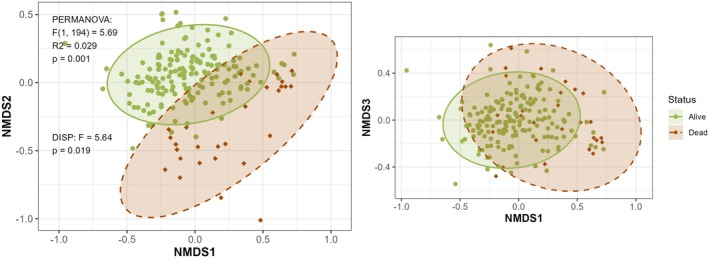
Ordination of fungal community structure grouped by bryophyte status (stress = 0.17). Ellipses represent the 95% confidence region for the group.

Bryophyte status had significant effects on fungal community diversity and composition, with living samples having higher diversity than necromass. Samples of living bryophyte tissue showed 30% higher taxonomic richness, 35% higher Chao1 richness, and 15% higher Shannon diversity than dead bryophytes (Figure 4a/b, Table A3). These diversity differences were accompanied by changes in fungal community composition and dispersion, with dead samples showing higher dispersion (Figures 4c, A3 and Table A4).

**FIGURE A4:**
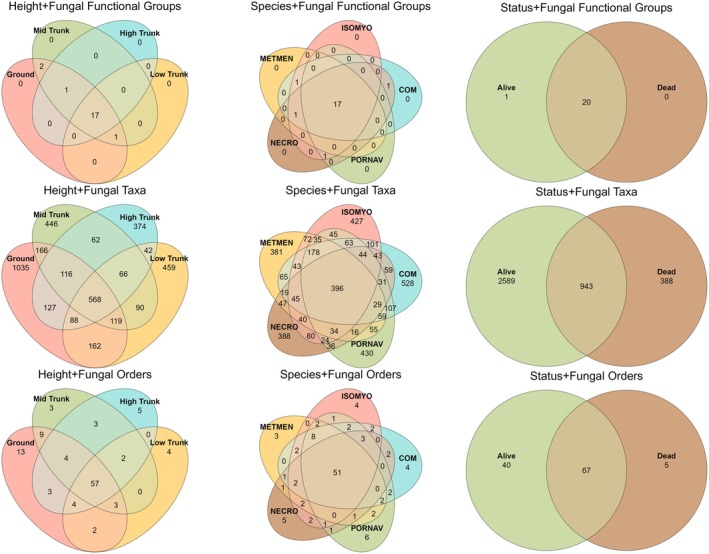
Venn diagrams showing overlapping ASVs, fungal orders, and functional groups between height categories.

**FIGURE A5:**
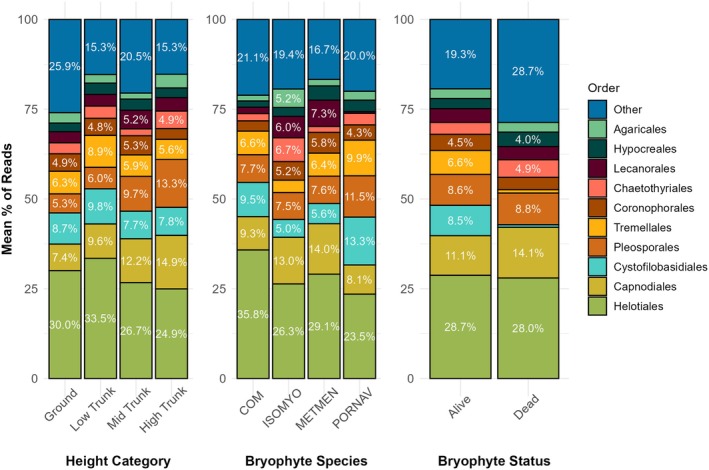
Stacked bar charts demonstrating the most abundant fungal orders and the average percentage of reads.

Fungal communities in living and dead bryophytes were functionally and taxonomically distinct. Fungi in the order Cystofilobasidiales were 12 times as abundant in living samples (*F* (1, 186) = 107.16, *t* = 10.35, *p* < 0.001), and fungi in Tremellales were 7.3 times as abundant in living samples (*F* (1, 194) = 75.97, *t* = 8.72, *p* < 0.001) (Figure A5, Table A9). Litter saprotrophs were 37% more abundant (*F* (1, 188) = 12.04, *t* = 3.47, *p* < 0.001) and soil saprotrophs were 51% more abundant in living samples (*F*(1, 187) = 13.51, *t* = 3.68, *p* < 0.001) (Figure 4d, Tables A10, A11). Wood saprotrophs, however, were 36% more abundant in dead bryophyte samples (*F* (1, 191) = 4.88, *t* = 2.21, *p* = 0.028) (Figure 4d, Table A10). Indicator species analyses identified 40 fungal genera that were associated with living bryophytes and 37 associated with dead bryophytes (Table A12). Notably, however, a majority of fungal taxa present in dead bryophyte samples were also found in living samples (Figure A4).

## 4 Discussion

Fine‐scale differences in fungal communities shape plant function, distribution, and health. To date, almost all work on plant‐fungal relationships has focused on ground‐rooted systems. Epiphyte communities contain a rich diversity of plant and microbial life and offer a way to examine the biotic and abiotic factors impacting fungal diversity and distribution at a fine spatial scale. Several studies offer comparisons between ground and canopy microbial communities in tropical forests (Gora et al. 2019; Looby et al. 2020; Pittl et al. 2010) and temperate forests (Rousk and Nadkarni 2009; Harrison et al. 2016). To our knowledge, however, few have directly compared ground‐rooted plant‐associated fungi with those in the canopy (Lehnert et al. 2017). Furthermore, only one study has examined the host specificity of fungi associated with epiphytic bryophytes (Cook and Taylor 2023), and there have been no investigations into epiphytic bryophyte‐associated microbes in temperate ecosystems. Our study is the first to simultaneously parse apart the role of height in the canopy, host species, and tissue decompositional stage in shaping fungal communities in a temperate forest ecosystem. Our results show striking differences between the ground and all canopy strata fungal communities, as well as strong differences between necromass and living bryophyte tissue. Epiphytic host species drove more subtle fungal community composition differences, demonstrating both a common “core microbiome” and some host specificity. Our taxonomic and functional analyses highlight the implications for the ecology of the host plants as well as the abiotic and biotic drivers of microbial communities.

Importantly, our functional analyses were limited by the sizable proportion of fungal ASVs that could not be assigned to a primary lifestyle. While this is consistent with many other publications that rely on functional databases, it nonetheless impacts the relative abundance of fungal lifestyles in our dataset (Tanunchai et al. 2023). Additionally, we may have biases from our ITS primers that could potentially limit our detection of some fungal taxa. Therefore, our interpretations of this data are conservative and reflect the available methods and the paucity of available information on fungal functional roles, especially for fungi that occur in underexplored locations like the canopy.

Rather than a full gradient from the ground to the upper canopy, we found the major differences in epiphyte‐associated fungal diversity and community composition between the ground samples and all tree‐collected samples. Unlike patterns of the epiphytic bryophytes themselves, who show strong differences in diversity, abundance, and composition across microsites within trees (Woods et al. 2019), sampled fungal communities within the tree were largely consistent. Contact with the forest floor, however, did engender distinct microbial communities. Fungal taxonomic richness and diversity were 34% and 17% higher, respectively, in bryophyte samples from the ground compared to all canopy‐level bryophyte samples. This broad pattern is consistent with previous studies comparing ground and epiphytic fungal communities in tropical forests (Looby et al. 2020; Gora et al. 2019). These patterns could be explained by the high moisture content (Parker et al. 1995; Gora et al. 2019; Looby et al. 2020), comparatively stable temperatures (Aubrey et al. 2013), and reduced sunlight exposure on the forest floor in comparison to the high temperature and aridity of the upper canopy (Parker et al. 1995; Gora et al. 2019). However, in one study of temperate canopy ecosystems, fungal growth was 3× higher in canopy soils than on the ground when measured using acetate incorporation into ergosterol (Rousk and Nadkarni 2009). This increased fungal growth was correlated with high organic matter content in *A. macrophyllum* canopy soils compared to the forest floor (Rousk and Nadkarni 2009). Another study comparing fungal community composition across a height gradient within coastal redwood canopies showed that redwood needles collected from different heights in the tree crown (2–108 m) harbored distinct assemblages of fungi, but fungal diversity was not associated with canopy height (Harrison et al. 2016). However, there were no ground‐level samples included in the analysis (Harrison et al. 2016).

In comparison to tropical forest canopies, there is much less microclimate variation within temperate *A. macrophyllum* canopies (e.g., Woods et al. 2019). The lack of a strong vertical microclimatic gradient within these trees could indicate that the fungal turnover we observed between the low trunk and high trunk is driven by structural factors, biotic interactions, or dispersal limitation (Woods et al. 2019; Gilbert 2005), rather than abiotic gradients like those found in tropical canopies. A much more in‐depth functional analysis (including characterizing abiotic tolerances of fungi) would be required to parse apart specific drivers of the functional turnover from the ground to the canopy; however, our results suggest an important biotic turnover along the vertical gradient. Saprotrophs, plant pathogens, and lichenized fungi all varied along the vertical gradient, independent of host epiphyte species. Epiphytic bryophyte communities vary according to canopy cover and substrate characteristics within the tree, and these fine‐scale structural differences could also drive the community composition of their associated microbes (Woods et al. 2019). The similar fungal community patterns observed across temperate and tropical ecosystems, despite their contrasting abiotic contexts, suggest that canopy‐ground differences in fungal diversity might be generalizable across a latitudinal gradient. Future studies should compare the canopy microbiome across ecosystems to assess the relative impact of biotic and abiotic factors on microbial diversity and composition.

Our results support biotic factors as a major driver of fungal communities within the canopy. We found that fungal community composition, community dispersion, and the relative abundance of different fungal taxa varied according to host bryophyte species. Of the 10 most common fungal orders in our samples, 7 showed significant differences in relative abundance among the host bryophyte species. The presence of certain fungal taxa on bryophytes could be driven by host characteristics such as leaf morphology, leaf chemistry, plant growth, and antimicrobial defenses (Li et al. 2020; Kembel and Mueller 2014; Apigo and Oono 2022; Mekuria et al. 2005). For example, lichen‐forming fungi were more abundant in samples of *M. menziesii* and *I. myosuroides* compared to *P. navicularis* and community samples. This is consistent with visible differences in lichen abundance across plots dominated by different bryophyte species that have been observed in the field, including a noticeably higher lichen abundance on *M. menziesii* patches (Spicer unpublished data). This difference could be due to the structure, active antifungal defenses, or even other fungi present in bryophyte species like *P. navicularis* , which harbored far more lichen parasites than *M. menziesii* . Certain bryophyte host species may be more readily colonized by fungi, contributing to the host specificity of certain fungal taxa (Davey et al. 2013). Fungal–plant pathogens, in particular, are highly host‐specific in vascular plants (Li et al. 2020), but it is unclear whether similar trends are present in nonvascular plant communities. The observed variation in fungal communities could also be underpinned by differences in the natural history of each focal species. As the only liverwort of our three focal species, *P. navicularis* may host a unique suite of fungi, as evidenced by the 26 fungal genera associated with *P. navicularis* . In contrast, indicator species analysis only identified 6 genera associated with *I. myosuroides* and 7 genera with *M. menziesii*. The associations between certain fungal taxa and bryophyte species could indicate cross‐taxon congruence, or the co‐variation of two taxonomically different groups in response to changes in spatial or environmental conditions. For example, if bryophyte communities serve as an indicator for fungal communities, this information could be used to optimize sampling efforts in biodiversity studies (Fanfarillo et al. 2024).

In addition to host plant characteristics, plant community differences can impact patterns in fungal community composition. Though significantly different in community composition, there was high overlap among bryophyte species, potentially indicating a core microbiome present across all bryophyte species. Out of 3920 unique fungal taxa, a core community of 31 taxa accounted for an average of 36.3% of all reads in living bryophyte samples, suggesting that the differences in community composition observed across species were more likely driven by rare fungal taxa. Additionally, the degree of host specialization observed among fungal taxa can vary depending on plant species abundance (Apigo and Oono 2022). More abundant plant species can harbor endophytes that occupy fewer plant species (i.e., fungi with higher host specificity) (Apigo and Oono 2022), and a high proportion of rare plant species can be associated with low host specificity among fungi (Cook and Taylor 2023). Compared to ground‐rooted communities, epiphyte systems contain a high diversity of low abundance plants, potentially driving a community of host‐generalist fungi (Cook and Taylor 2023; Woods et al. 2019). We selected our three focal bryophyte species because of their presence across a height gradient, but future studies should consider sampling fungi from a wider range of epiphytic plant species to assess the extent of host specificity across a height gradient. The relative abundance of core fungal taxa was consistent across bryophyte species, but ground samples had a markedly lower relative abundance of core taxa (24%) compared to tree‐level samples (40%). These findings support the existence of both a core microbiome and a majority of less common taxa across different bryophyte species within the canopy.

In addition to the opportunity to vary across living epiphytic bryophyte host species, fungal community differentiation could occur as host plants die. Independent of bryophyte species, living bryophyte samples contained higher fungal diversity and taxonomic richness than samples of senescent bryophyte tissue, indicating a truly biotic component to fungal–plant interactions. Although we did not separate fungal samples within versus outside bryophyte tissues, senescent bryophytes were clearly a more homogeneous habitat for fungi than the living plants, with 30% lower species richness and 15% lower diversity. Similar trends in diversity were observed in a tropical forest, with living bryophytes showing higher fungal OTU richness than dead bryophytes, outer bark, and inner bark (Cook et al. 2022). A study of ground‐occurring bryophytes in a boreal forest showed a higher predicted OTU richness in living bryophytes compared to senescent bryophyte tissue, though the relative abundances of fungal taxa were seasonally variable (Davey et al. 2012). We observed differences in the relative abundances of certain fungal orders and functional groups associated with living and senescent bryophytes. For example, fungi in the order Cystofilobasidiales were more abundant in living bryophytes than dead bryophytes, and the vast majority of fungal taxa in this order were identified as plant pathogens. However, the relative abundance of plant pathogens was not different between living and dead samples. These contrasting results could indicate that certain fungal taxa may have varying success in colonizing living bryophytes. In one study, certain saprotrophic taxa were, notably, more frequent and abundant in senescent than photosynthetic tissue (Davey et al. 2013). Surprisingly, our results indicated a higher relative abundance of certain saprotrophic groups associated with living bryophytes than dead bryophyte tissue, including litter saprotrophs and soil saprotrophs. Fungi in the order Tremellales, which were more abundant in living samples, are typically associated with a soil saprotrophic lifestyle.

Contrary to our expectations, and despite their lower diversity and taxonomic richness, senescent bryophytes had a higher dispersion of community composition. This could be attributed to temporal shifts, as different stages of decomposition could be associated with shifts in the fungal community. Additionally, the lack of active defense mechanisms of senescent tissue could provide an opportunity for a wide range of fungal taxa to colonize it. Nevertheless, living bryophytes harbored significantly higher fungal diversity and richness, potentially owing to the complex structure, tissue heterogeneity, and active antifungal defenses of living bryophytes (Votintseva 2007; Orlovich et al. 2013; Cook et al. 2022). Together, these factors may provide opportunities for high fungal niche differentiation, and thus high diversity. It should be noted, however, that fungal nutritional modes are not necessarily static. The ecological role of a bryophyte‐associated fungal species could depend on whether its host is alive or dead, as fungi that are endophytic in a living host may switch to a saprotrophic habit upon the death of that host (Chen et al. 2022). Furthermore, most bryosymbiotic fungi in Ascomycota appear to have evolved from saprotrophic ancestors, with this shift occurring multiple times and even within genera (Stenroos et al. 2010). It is possible that the enzymatic control mechanisms of these saprobic fungi, originally involved in wood and soil decay, later allowed for the colonization of bryophyte substrates (Stenroos et al. 2010). This could provide some evolutionary context for the high proportion of fungal taxa that were assigned to a saprotrophic lifestyle in our study, as bryosymbiotic fungi appear more closely related to saprobes than to endophytic fungi of vascular plants. The paucity of information on ecological relationships between fungi and bryophytes, especially in the canopy, limits our understanding of the dynamics underpinning the observed functional shifts between living and dead samples.

Fungal community diversity and composition are highly complex. Epiphytic bryophytes provide a useful system for studying fungal community dynamics because they represent a wide variety of ecological niches and microhabitats at a small spatial scale and live in close association with each other, providing ample opportunities for both biotic and abiotic structuring of communities. Vertical position and substrate characteristics provide opportunities for niche differentiation and complex species interactions, potentially driving microbial diversity in epiphyte‐associated communities. Previous work on ground‐rooted communities indicates that the distribution and arrangement of fungi can be due to factors such as host specificity, dispersal limitation, and different life strategies, and our work suggests that these factors are likely to be at play in epiphyte communities as well. Still, the field of epiphyte‐microbe interactions is largely unexplored. Our findings suggest that there is a core community of fungi present within the canopy, with fungal taxa that vary in relative abundance depending on canopy height and bryophyte species. Fungal diversity was higher on the ground than in the canopy among living bryophytes, and higher among living bryophytes than senesced bryophytes. These results are novel and demonstrate the combined effects of abiotic and biotic drivers of fungal community assembly within the vertical strata of a temperate forest. Examining the microbial communities that interact with nonvascular epiphytes could eventually have upstream implications in forest dynamics, epiphyte evolution, and plantmicrobe interactions.

## Author Contributions

**Laurel Renee Humphreys:** conceptualization (equal), data curation (supporting), formal analysis (lead), funding acquisition (supporting), investigation (equal), methodology (equal), project administration (supporting), resources (supporting), visualization (lead), writing – original draft (lead), writing – review and editing (supporting). **Jane M. Lucas:** conceptualization (equal), data curation (lead), formal analysis (supporting), funding acquisition (supporting), investigation (equal), methodology (equal), project administration (equal), resources (equal), software (lead), supervision (supporting), writing – review and editing (equal). **Michelle Elise Spicer:** conceptualization (equal), formal analysis (supporting), funding acquisition (lead), investigation (equal), methodology (equal), project administration (lead), resources (lead), supervision (lead), validation (lead), writing – review and editing (lead).

## Disclosure

Statement on Inclusion: All authors of this study are based in the country where the research was conducted. The experiment was established in collaboration with a locally based scientist, ensuring that local expertise and knowledge informed the study design.

## Conflicts of Interest

The authors declare no conflicts of interest.

## Data Availability

All data supporting the findings of this study are openly available in the Dryad repository at https://doi.org/10.5061/dryad.rr4xgxdjj (Humphreys et al. 2025).

## Appendix A

**TABLE A1:** Location of each *Acer macrophyllum* tree involved in our study, and the approximate height of each vertical location where samples were collected.

Tree	**Height (m)** low trunk (V1)	**Height (m)** mid trunk (V2)	**Height (m)** high trunk (V3)
1	0	5.4	10.5
2	0.8	7.2	16.6
3	0.5	3.8	11.6
4	0.9	5.9	13.9
5	1	4.5	14
6	1.1	6	15.8
7	1.9	3.9	14.6
8	0.7	5.1	10.5
9	1.3	7.1	17.8
10	1.1	7	15
11	0.6	4.2	9.4
12	0.9	6.3	17.5

**TABLE A2:** Summary of sequencing data across bryophyte substrate types and height categories before and after cleaning and rarefaction. This includes the total number of samples in each category, the number of sequencing reads, and the mean number of sequencing reads per sample.

	Sample category	Before cleaning and rarefaction			After cleaning and rarefaction		
		*N*	No. of reads	Mean no. of reads per sample	*N*	No. of reads	No. of reads per sample
Height	Ground (T0)	60	1,517,176	25,286	51	105,672	2072
	Low trunk (V1)	59	1,088,819	18,455	46	95,312	2072
	Mid trunk (V2)	60	1,256,886	20,948	50	103,600	2072
	High trunk (V3)	59	1,075,976	18,237	49	101,528	2072
Species	IsoMyo	47	1,038,660	22,099	39	80,808	2072
	MetMen	47	1,038,535	22,096	42	87,024	2072
	PorNav	48	1,037,886	21,623	43	89,096	2072
	Community	48	1,147,279	23,902	43	89,096	2072
Status	Alive	190	4,262,360	22,433	167	346,024	2072
	Dead	48	676,497	14,094	29	60,088	2072
**Total**		**238**	**4,939,307**	**20,753**	**196**	**406,112**	**2072**

**TABLE A3:** Diversity data for fungal taxa and functional groups. All values represent the averages within each group (±SE). Beta diversity reflects the average dissimilarity of each sample from the group mean, using Bray–Curtis dissimilarity. Superscripts denote similar values.

		*N*	Fungal ASVs				Functional groups		
			Richness	Chao1	Shannon	Beta	Richness	Shannon	Beta
Height (alive samples only)	T0	42	168^a^ (10.2)	218.5^a^ (15.5)	3.56^a^ (0.13)	0.87 (0.01)	12.1 (0.3)	1.68 (0.04)	0.37 (0.02)
	V1	40	132.0^b^ (8.7)	173.8^b^ (14.3)	3.03^b^ (0.12)	0.81 (0.01)	10 (0.3)	1.56 (0.05)	0.37 (0.02)
	V2	43	125.9^b^ (7.9)	165.6^b^ (11.4)	3.05^b^ (0.11)	0.87 (0.01)	9.9 (0.3)	1.52 (0.05)	0.41 (0.02)
	V3	42	119.1^b^ (7)	148.2^b^ (9.2)	3.07^b^ (0.12)	0.87 (0.01)	9.7 (0.3)	1.48 (0.05)	0.41 (0.02)
Bryophyte Species (alive samples only)	IsoMyo	39	133.3^a^ (8.2)	170.5^a^ (11.8)	3.22^a^ (0.12)	0.93 (0)	10.4 (0.3)	1.6 (0.05)	0.4 (0.02)
	MetMen	42	126.9^a^ (7.7)	162.6^a^ (11.2)	3.13^a^ (0.11)	0.85 (0.01)	9.8 (0.3)	1.51 (0.05)	0.41 (0.02)
	PorNav	43	139.1^a^ (9.2)	182^a^ (13.9)	3.12^a^ (0.14)	0.84 (0.01)	10.4 (0.4)	1.55 (0.05)	0.37 (0.02)
	Community	43	145.2^a^ (10.2)	190^a^ (15.5)	3.26^a^ (0.13)	0.84 (0.01)	11 (0.4)	1.59 (0.04)	0.38 (0.02)
Status	Alive	167	136.2^a^ (4.5)	176.5^a^ (6.7)	3.18^a^ (0.06)	0.88 (0)	10.4 (0.2)	1.56 (0.02)	0.51 (0.03)
	Dead	29	104.9^b^ (9.1)	130.9^b^ (12.8)	2.77^b^ (0.17)	0.96 (0)	10 (0.4)	1.49 (0.08)	0.40 (0.01)

Effect	Response	NumDF	DegDF	*F*	*p*	Pairwise
Height	Richness	3	150.08	7.3597	< 0.001	T0 > V1, T0 > V2, T0 > V3
Height	Chao1	3	150.17	6.1992	< 0.001	T0 > V1, T0 > V2, T0 > V3
Height	Shannon	3	150.38	4.5166	0.005	T0 > V1, T0 > V2, T0 > V3
Status	Richness	1	183.09	11.344	< 0.001	Alive > Dead
Status	Chao1	1	183.19	10.5403	0.001	Alive > Dead
Status	Shannon	1	183.92	8.7368	0.003	Alive > Dead

**TABLE A4:** PERMANOVA statistics. Fungal community differences according to bryophyte height, species, and status.

Effect	Df	SumOfSqs	*R* ^2^	*F*	Pr(>*F*)	Pairwise
Height	3	3.204	0.05017	2.8696	0.001 ***	T0/V1***, T0/V2***, T0/V3***, V1/V2*, V1/V3***, V2/V3*
Species	3	3.155	0.04939	2.8232	0.001 ***	COM/ISOMYO***, COM/METMEN***, COM/PORNAV**, ISOMYO/METMEN**, ISOMYO/PORNAV***, METMEN/PORNAV***
Total	166	63.868	1.00000			

Effect	Df	SumOfSqs	*R* ^2^	F	Pr(>*F*)
Status	1	2.225	0.02851	5.6928	**0.001*****
Total	195	78.050	1.00000		

*Note:* Asterisks denote significant *p*‐values. Example of one PERMANOVA model and pairwise comparisons: permanova <‐ adonis2(sub.alive ~ Height, data = meta.alive, permutations = 999, method = “bray”). pairwise.adonis2(sub.alive ~ Height, data = meta.alive, sim.method = “bray”, perm = 999). Model for Status, using data for both living and dead bryophyte samples: permanova <− adonis2(t(otu) ~ Status, data = meta, permutations = 999, method = “bray”). pairwise.adonis2(t(otu)~ Status, data = meta, sim.method = “bray”, perm = 999).

**TABLE A5:** Mean percentages of total sequences (±SE) in a sample within each height category (alive samples only) for the 10 most abundant orders of fungi, with statistical results of comparisons after logit transformation of relative abundance. Bold text indicates orders representing, on average, more than 5% of total reads for a sample at that height.

Order	Ground (*n* = 42)	Low Trunk (*n* = 40)	Mid Trunk (*n* = 43)	High Trunk (*n* = 42)	Mean Sq	*F*	*p*
Agaricales	2.9 (0.9)	2.4 (0.5)	1.7 (0.6)	3.8 (2.3)	10.79	1.29	0.280
Capnodiales	**7.4 (0.9)**	**9.6 (1.3)**	**12.2 (2.2)**	**14.9 (2.3)**	3.08	2.91	**0.036 ***
Chaetothyriales	3.1 (0.6)	3.4 (0.9)	1.9 (0.4)	4.9 (1.8)	1.05	0.12	0.946
Coronophorales	4.9 (1.5)	4.8 (1.3)	**5.3 (1.2)**	3 (1.1)	36.61	2.17	0.094
Cystofilobasidiales	**8.8 (1.4)**	**9.8 (1.2)**	**7.7 (1.5)**	**7.8 (1.9)**	15.22	3.93	**0.009 ****
Helotiales	**30.0 (2.7)**	**33.5 (3.5)**	**26.7 (3.0)**	**24.9 (2.7)**	2.50	2.35	0.075
Hypocreales	2.4 (0.5)	3.2 (0.7)	3.1 (0.6)	2.7 (0.6)	11.33	0.81	0.491
Lecanorales	3.1 (1.4)	3.2 (1.3)	**5.2 (1.9)**	3.7 (1.1)	53.28	2.33	0.076
Pleosporales	**5.3 (0.9)**	**6 (0.8)**	**9.7 (1.9)**	**13.3 (2.8)**	5.06	2.82	**0.041 ***
Tremellales	**6.3 (0.7)**	**8.9 (1.4)**	**5.9 (1.2)**	**5.6 (1)**	8.49	1.79	0.151

*Note:* Asterisks denote significant *p*‐values. Example of one linear model: model <‐ lmer(logit_Agaricales ~ Height + Species + (1 | TreeID), data = ord.rel.alive).

**TABLE A6:** Mean percentages of total sequences (±SE) in a sample within each height category (alive samples only) for fungal functional groups, with statistical results of comparisons after logit transformation of relative abundance. Functional groups with less than 100 total reads are not reported. Bold text indicates orders representing, on average, more than 5% of total reads for a sample at that height. “^” denotes significant association in Indicator species analysis.

Functional type	Functional group	Ground	Low trunk	Mid trunk	High trunk	Mean Sq	*F*	*p*
Saprotroph	Dung saprotroph	0.2 (0.1)^	0 (0)	0 (0)	0 (0)	20.19	3.48	**0.017***
	Litter saprotroph	**19.1 (1.7)^**	**13.0 (1.5)**	**12.7 (1.4)**	**14.7 (1.8)**	2.06	2.93	**0.036***
	Pollen saprotroph	0.5 (0.2)^	0.1 (0)	0.1 (0)	0.1 (0)	124.45	6.35	**< 0.001*****
	Soil saprotroph	**11.5 (1.1)**	**12.6 (1.5)**	**8.0 (1.1)**	**10.6 (1.9)**	2.73	2.70	**0.048***
	Wood saprotroph	**19.7 (3)^**	**10.6 (1.5)**	**18.0 (2.5)**	**12.6 (2)**	5.28	4.18	**0.007****
	Unspecified saprotroph	**15.7 (1.4)**	**34 (3.0)**	**26.8 (3)**	**30.68 (3)**	9.00	9.36	**< 0.001*****
Pathogens and parasites	Algal parasite	0.2 (0)^	0 (0)	0 (0)	0 (0)	167.59	11.22	**< 0.001*****
	Animal parasite	1.2 (0.3)	1.8 (0.7)	1.3 (0.3)	0.8 (0.2)	13.52	0.83	0.479
	Lichen parasite	2.1 (0.4)	0.8 (0.2)	2.3 (0.9)	1.3 (0.5)	86.59	4.88	**0.003****
	Mycoparasite	0.8 (0.2)	0.5 (0.1)	0.3 (0.1)	0.9 (0.5)	67.76	3.17	**0.026**
	Plant pathogen	**19.2 (1.7)**	**18.3 (1.5)**	**19 (1.9)**	**19.1 (2.6)**	0.1	0.11	0.95
Symbionts	Ectomycorrhizal	0.3 (0.3)^	0 (0)	0 (0)	0 (0)	40.87	2.81	**0.041 ***
	Epiphyte	0.3 (0.2)^	0 (0)	0 (0)	0 (0)	87.92	5.24	**0.002 ****
	Foliar endophyte	0.2 (0.1)	0 (0)	0 (0)	0 (0)	37.72	3.15	**0.027 ***
	Root endophyte	4.6 (1.3)	2.6 (1.1)	3.1 (1.4)	1.7 (0.5)	116.46	4.56	**0.004 ****
Lichenized	Lichenized	4.6 (1.7)	**5.5 (1.7)**	**8.2 (2.1)**	**7.3 (2)**	8.38	0.75	0.524

*Note:* Asterisks denote significant *p*‐values. Example of one linear model: model <‐ lmer(logit_rel_wood_saprotroph ~ Height + Species + (1 | TreeID), data = subset.alive.fun).

**TABLE A7:** Mean percentages of total sequences (±SE) in a sample from each bryophyte species for the 10 most abundant orders of fungi, with statistical results of comparisons after logit transformation of relative abundance. Bold text indicates orders representing, on average, more than 5% of total reads for a sample of that bryophyte species.

Order	ISOMYO	METMEN	PORNAV	COM	Mean Sq	*F*	*p*
Agaricales	5.2 (2.5)	1.8 (0.8)	2.5 (0.7)	1.6 (0.3)	18.88	2.25	0.084
Capnodiales	**13.0 (2)**	**14.0 (2.0)**	**8.1 (1.6)**	**9.3 (1.5)**	3.25	3.07	**0.029***
Chaetothyriales	**6.7 (2)**	1.7 (0.3)	3.2 (0.7)	2.0 (0.4)	25.32	2.98	**0.033***
Coronophorales	**5.2 (1.4)**	**5.8 (1.6)**	4.3 (1.2)	2.9 (0.9)	7.05	0.42	0.741
Cystofilobasidiales	5 (0.8)	**5.6 (1.1)**	**13.3 (2)**	**9.5 (1.6)**	14.74	3.81	**0.011***
Helotiales	**26.3 (2.5)**	**29.1 (3.3)**	**17.9 (2.7)**	**20.4 (1.7)**	3.94	3.69	**0.013***
Hypocreales	2.5 (0.4)	4 (0.8)	3.2 (0.8)	1.7 (0.3)	15.17	1.08	0.358
Lecanorales	**6 (1.7)**	**7.3 (2.0)**	0.5 (0.1)	1.8 (1.0)	237.99	10.44	**< 0.001*****
Pleosporales	**7.5 (2.1)**	**7.6 (1.4)**	**11.5 (2.3)**	**7.7 (1.5)**	4.79	2.67	**0.049***
Tremellales	3.4 (0.6)	**6.4 (1.3)**	**9.9 (1.2)**	**6.6 (0.8)**	30.96	6.53	**< 0.001*****

*Note:* Asterisks denote significant *p*‐values. Example of one linear model: model <‐ lmer(logit_Agaricales ~ Height + Species + (1 | TreeID), data = ord.rel.alive).

**TABLE A8:** Mean percentages of total sequences (±SE) in a sample from each bryophyte species for fungal functional groups, with statistical results of comparisons after logit transformation of relative abundance. Functional groups with less than 100 total reads are not reported. Bold text indicates orders representing, on average, more than 5% of total reads for a sample from that bryophyte species. “^” denotes significant association in Indicator species analysis.

Functional type	Functional group	ISOMYO	METMEN	PORNAV	COM	Mean Sq	*F*	*p*
Saprotroph	Dung saprotroph	0.2 (0.2)	0 (0)	0 (0)	0 (0)	9.71	1.67	0.17
	Litter saprotroph	**16 (1.6)**	**12.2 (1.7)**	**15.2 (1.6)**	**16.3 (1.8)**	1.42	2.02	0.11
	Pollen saprotroph	0.1 (0)	0.3 (0.2)	0.1 (0)	0.2 (0)	58.65	2.99	**0.032***
	Soil saprotroph	**10.9 (2.1)**	**8.6 (1.3)**	**13 (1.4)**	**10.0 (0.9)**	2.56	2.54	0.059
	Wood saprotroph	**14.5 (2)**	**19.9 (3.0)**	**13.5 (2.3)**	**13.4 (1.8)**	1.89	1.5	0.217
	Unspecified saprotroph	**19.2 (2.7)**	**27.1 (2.7)**	**28.1 (2.9)**	**31.7 (2.9)^**	4.80	4.99	**0.003****
Pathogens and parasites	Algal parasite	0.1 (0)	0.1 (0)	0.1 (0)	0 (0)	16.73	1.12	0.34
	Animal parasite	0.9 (0.2)	1.2 (2.2)	1.9 (0.7)	1 (0.2)	2.98	0.18	0.908
	Lichen parasite	1.4 (0.4)	0.7 (0.3)	2.2 (0.8)	2.3 (0.5)	108.77	6.13	**< 0.001*****
	Mycoparasite	0.5 (0.1)	0.3 (0.1)	0.6 (0.2)	1.0 (0.5)	13.13	0.62	0.606
	Plant pathogen	**21.7 (2.8)**	**16.4 (1.6)**	**11.6 (0.1)**	**15.4 (1.4)**	2.08	2.33	0.077
Symbionts	Ectomycorrhizal	0.3 (0.3)	0.1 (0)	0.1 (0)	0 (0)	11.08	0.76	0.517
	Epiphyte	0.1 (0)	0.2 (0.15)	0 (0)	0.1 (0)	15.47	0.92	0.432
	Foliar endophyte	0.1 (0)	0 (0)	0 (0)	0.2 (0.1)	25.20	2.10	0.102
	Root endophyte	**3.7 (1.1)**	**2 (1.2)**	**1.0 (0.2)**	**5.4 (1.5)**	63.75	2.5	0.062
Lichenized	Lichenized	**10.5 (2.3)**	**10.77 (2.5)^**	**1.8 (0.4)**	**3.1 (1.3)**	123.76	11.07	**< 0.001*****

*Note:* Asterisks denote significant *p*‐values. Ex. model <− lmer(logit_rel_wood_saprotroph ~ Height * Species + (1 | TreeID), data = subset.alive.fun).

**TABLE A9:** Mean percentages of total sequences (±SE) in a sample from each bryophyte status for the 10 most abundant orders of fungi, with statistical results of comparisons after logit transformation of relative abundance. Bold text indicates orders representing, on average, more than 5% of total reads for a sample of that category.

Order	Alive (*n* = 167)	Dead (*n* = 29)	Mean Sq	*F*	*p*
Agaricales	2.7 (0.6)	2.7 (0.9)	43.46	4.04	**0.046***
Capnodiales	**11.1 (0.9)**	**14.1 (2.5)**	0.88	0.69	0.405
Chaetothyriales	3.3 (0.5)	4.9 (2.3)	0.43	0.05	0.824
Coronophorales	4.5 (0.6)	3.4 (1.4)	185.42	9.69	**0.002****
Cystofilobasidiales	**8.5 (0.8)**	0.7 (0.3)	775.94	107.16	**< 0.001*****
Helotiales	**28.7 (1.5)**	**28 (4.1)**	0.25	0.19	0.658
Hypocreales	2.8 (0.3)	4.0 (1.8)	13.57	0.96	0.328
Lecanorales	3.8 (0.7)	3.7 (1.4)	95.30	3.35	0.069
Pleosporales	**8.6 (0.9)**	**8.8 (3.3)**	28.19	9.69	**0.002****
Tremellales	**6.6 (0.5)**	0.9 (0.2)	690.50	75.97	**< 0.001*****

*Note:* Asterisks denote significant *p*‐values. Example of one linear model: model <‐ lmer(logit_Agaricales ~ Status + (1 | TreeID), data = ord.rel.meta).

**TABLE A10:** Mean percentages of total sequences (±SE) in a sample from each bryophyte status for fungal functional groups, with statistical results of comparisons after logit transformation of relative abundance. Functional groups with less than 100 total reads are not reported. Bold text indicates orders representing, on average, more than 5% of total reads for a sample from that category. “^” denotes significant association in Indicator species analysis.

Functional type	Functional group	Alive	Dead	Mean Sq	*F*	*p*
Saprotroph	Dung saprotroph	0 (0)	0.2 (0.1)	20.08	2.75	0.099
	Litter saprotroph	**14.9 (0.8)^**	**10.8 (1.9)**	10.56	12.04	**< 0.001*****
	Pollen saprotroph	0.2 (0.1)	0.1 (0)	90.01	4.18	**0.042***
	Soil saprotroph	**10.6 (0.7)**	**7.0 (2.4)**	16.55	13.51	**< 0.001*****
	Wood saprotroph	**15.3 (1.2)**	**20.9 (3.0)**	6.51	4.88	**0.028***
	Unspecified saprotroph	**26.7 (1.4)^**	**16.0 (3.8)**	26.45	19.31	**< 0.001*****
Pathogens and parasites	Algal parasite	0.1 (0)	0 (0)	12.55	0.73	0.394
	Animal parasite	1.3 (0.2)	2.1 (0.8)	5.04	0.29	0.593
	Lichen parasite	1.7 (0.3)	1.6 (0.8)	59.1	2.61	0.108
	Mycoparasite	0.6 (0.1)	1.8 (0.6)	24.38	1.01	0.317
	Plant pathogen	**18.9 (1)**	**21.7 (3.6)**	0.08	0.08	0.783
Symbionts	Ectomycorrhizal	0.1 (0.1)	0.1 (0)	66.47	4.08	**0.045***
	Epiphyte	0.1 (0)	0.2 (0.2)	73.75	4.37	**0.038***
	Foliar endophyte	0.1 (0)	0.3 (0.2)	0.72	0.05	0.818
	Root endophyte	3.0 (0.6)	**6.1 (3.1)**	0.81	0.03	0.867
Lichenized	Lichenized	**6.4 (0.9)**	**11.0 (2.7)**	26.95	2.02	0.157

*Note:* Asterisks denote significant *p*‐values. Ex. model <− lmer(logit_rel_wood_saprotroph ~ Status + (1 | TreeID), data = lifestyle.rel).

**TABLE A11:** Indicator species analysis for functional data. Total number of functional groups: 21.

Group	Functional group	stat	*p*
Height: Ground (T0)	wood_saprotroph	0.589	0.035*
	pollen_saprotroph	0.586	0.005**
	algal_parasite	0.583	0.005**
	litter_saprotroph	0.577	0.050*
	ectomycorrhizal	0.503	0.015*
	epiphyte	0.478	0.010**
	dung_saprotroph	0.452	0.005**
Species: METMEN	lichenized	0.558	0.04*
Species: Community	unspecified_saprotroph	0.53	0.025*
Status: Alive	unspecified_saprotroph	0.820	0.005**
	litter_saprotroph	0.813	0.005**
Status: Dead	arbuscular_mycorrhizal	0.366	0.005**

*Note:* Asterisks denote significant *p*‐values.

**TABLE A12:** Indicator species analysis for taxonomic data. Total number of genera: 559.

Group	Stat	*p*
**Height**		
*Ground (T0)—59 genera*		
Gorgomyces	0.773	0.005**
Alatospora	0.734	0.005**
Sclerotinia	0.729	0.005**
Calloria	0.654	0.005**
Calycellina	0.642	0.005**
Uredinophila	0.623	0.005**
Tetracladium	0.594	0.015*
Mycoarthris	0.582	0.005**
Scirrhia	0.579	0.010**
Chrysozyma	0.577	0.005**
Retiarius	0.570	0.005**
Lophodermium	0.564	0.005**
Mycodidymella	0.557	0.005**
Rhizophydium	0.553	0.005**
Micarea	0.550	0.005**
Bryoglossum	0.549	0.005**
Fusicolla	0.544	0.005**
Leucosporidium	0.544	0.030*
Curvibasidium	0.518	0.010**
Bannozyma	0.517	0.005**
Yamadamyces	0.512	0.005**
Nigrograna	0.499	0.010**
Claussenomyces	0.495	0.005**
Coleophoma	0.482	0.020*
Piskurozyma	0.476	0.025*
Pseudoanungitea	0.474	0.005**
Neosetophoma	0.469	0.005**
Hymenoscyphus	0.468	0.005**
Taphrina	0.419	0.045*
Sclerococcum	0.417	0.005**
Murilentithecium	0.407	0.005**
Fellozyma	0.388	0.010**
Fuscopannaria	0.379	0.005**
Hyaloscypha	0.370	0.005**
Apodus	0.366	0.005**
Leptosphaeria	0.359	0.010**
Clavulinopsis	0.357	0.010**
Psathyrella	0.357	0.010**
Pycnora	0.342	0.005**
Plagiostoma	0.338	0.005**
Fuscostagonospora	0.326	0.015*
Cryptococcus	0.317	0.045*
Rosettozyma	0.317	0.010**
Pilidium	0.313	0.005**
Varicellaria	0.310	0.010**
Kondoa	0.308	0.045*
Gyoerffyella	0.306	0.035*
Trichomerium	0.303	0.040*
Botrytis	0.289	0.040*
Pseudoclathrosphaerina	0.288	0.030*
Mycoceros	0.280	0.015*
Galerina	0.280	0.020*
Pseudosigmoidea	0.280	0.025*
Verrucaria	0.280	0.025*
Scleropezicula	0.278	0.045*
Venturiocistella	0.262	0.025*
Rhizosphaera	0.259	0.025*
Herpotrichia	0.243	0.050*
Pyrenochaetopsis	0.243	0.050*
*Low Trunk (V1)—14 genera*		
Polyphilus	0.655	0.005**
Ramularia	0.634	0.010**
Simplicillium	0.557	0.010**
Tricellula	0.551	0.035*
Agonimia	0.547	0.005**
Coprinellus	0.465	0.005**
Arbusculina	0.428	0.010**
Vibrissea	0.348	0.010**
Friedmanniomyces	0.283	0.010**
Leptobacillium	0.273	0.025*
Thoreauomyces	0.258	0.045*
Phenoliferia	0.255	0.030*
Tetrapyrgos	0.209	0.050*
Verticimonosporium	0.209	0.050*
*Mid Trunk (V2)—5 genera*		
Acrodontium	0.668	0.005**
Lepraria	0.511	0.045*
Sistotrema	0.500	0.050*
Psoroglaena	0.471	0.035*
Athelia	0.238	0.050*
*High Trunk (V3)—7 genera*		
Cladosporium	0.698	0.005**
Xenodevriesia	0.600	0.010**
Clavidisculum	0.483	0.010**
Pectenia	0.468	0.015*
Fellomyces	0.288	0.030*
Batcheloromyces	0.286	0.005**
Schizophyllum	0.247	0.030*
**Bryophyte species**		
*Community—18 genera*		
Polyphilus	0.594	0.005**
Chrysozyma	0.567	0.005**
Epithamnolia	0.548	0.020*
Rhodosporidiobolus	0.548	0.010**
Taphrina	0.497	0.005**
Fellozyma	0.477	0.005**
Pyrenopeziza	0.433	0.030*
Yamadamyces	0.407	0.010**
Geomyces	0.393	0.020*
Hamamotoa	0.386	0.005**
Heitmania	0.342	0.010**
Thoreauomyces	0.319	0.015*
Phragmocephala	0.314	0.050*
Colacogloea	0.305	0.015*
Botrytis	0.291	0.020*
Oberwinklerozyma	0.286	0.045*
Aureobasidium	0.264	0.035*
Entyloma	0.264	0.030*
*ISOMYO—6 genera*		
Infundichalara	0.577	0.010**
Gyrographa	0.470	0.005**
Micarea	0.455	0.035*
Claussenomyces	0.387	0.030*
Byssoloma	0.332	0.020*
Paracladophialophora	0.226	0.050*
*METMEN—7 genera*		
Lepraria	0.568	0.005**
Chlamydocillium	0.563	0.010**
Incertomyces	0.554	0.005**
Psoroglaena	0.483	0.015*
Acremonium	0.427	0.035*
Beauveria	0.391	0.045*
Paraleptosphaeria	0.263	0.050*
*PORNAV—26 genera*		
Mrakia	0.603	0.005**
Heterobasidion	0.601	0.005**
Vishniacozyma	0.598	0.005**
Itersonilia	0.592	0.015*
Tetracladium	0.579	0.005**
Dioszegia	0.542	0.005**
Stagonospora	0.542	0.005**
Cistella	0.535	0.005**
Calloria	0.532	0.025*
Angustimassarina	0.505	0.010**
Leucosporidium	0.505	0.040*
Hannaella	0.490	0.010**
Nigrograna	0.478	0.010**
Boeremia	0.465	0.015*
Piskurozyma	0.455	0.020*
Tubulicrinis	0.444	0.030*
Clavidisculum	0.422	0.010**
Peltigera	0.407	0.045*
Prosthemium	0.349	0.020*
Skvortzovia	0.348	0.005**
Pholiota	0.341	0.010**
Hypholoma	0.340	0.035*
Fonsecazyma	0.310	0.020*
Fuscostagonospora	0.290	0.050*
Bryochiton	0.286	0.035*
Bisporella	0.245	0.045*
**Status**		
*Alive—40 genera*		
Vishniacozyma	0.949	0.005**
Itersonilia	0.947	0.005**
Mrakia	0.944	0.005**
Rhodosporidiobolus	0.890	0.005**
Ramularia	0.882	0.005**
Heterobasidion	0.878	0.005**
Cladosporium	0.875	0.005**
Pleurotus	0.864	0.005**
Sclerotinia	0.856	0.005**
Calloria	0.819	0.005**
Ganoderma	0.807	0.030*
Leucosporidium	0.792	0.005**
Microdochium	0.783	0.030*
Epithamnolia	0.782	0.005**
Tetracladium	0.761	0.005**
Cistella	0.736	0.005**
Scirrhia	0.688	0.010**
Trametes	0.681	0.025*
Chrysozyma	0.677	0.005**
Piskurozyma	0.667	0.005**
Sistotrema	0.655	0.030*
Uredinophila	0.653	0.015*
Fusarium	0.650	0.010**
Tubulicrinis	0.647	0.010**
Tricellula	0.647	0.015*
Mycodidymella	0.645	0.020*
Didymella	0.645	0.005**
Stagonospora	0.624	0.005**
Hannaella	0.613	0.020*
Nigrograna	0.597	0.025*
Curvibasidium	0.594	0.040*
Dioszegia	0.588	0.025*
Taphrina	0.574	0.010**
Retiarius	0.573	0.030*
Amphosoma	0.547	0.015*
Angustimassarina	0.547	0.040*
Cystofilobasidium	0.485	0.045*
Neosetophoma	0.479	0.045*
Fellozyma	0.445	0.025*
Hamamotoa	0.442	0.025*
*Dead—37 genera*		
Xenodevriesia	0.783	0.030*
Pectenia	0.776	0.005**
Serendipita	0.662	0.015*
Mortierella	0.639	0.005**
Sclerococcum	0.575	0.005**
Pezoloma	0.504	0.005**
Teratosphaeria	0.486	0.005**
Bryoglossum	0.466	0.005**
Phlogicylindrium	0.414	0.010**
Rhizoctonia	0.410	0.005**
Acrostalagmus	0.400	0.005**
Pseudoanungitea	0.374	0.040*
Hyphodontia	0.361	0.020*
Ilyonectria	0.357	0.005**
Atractospora	0.357	0.020*
Amicodisca	0.355	0.035*
Thelonectria	0.346	0.010**
Lophiotrema	0.341	0.040*
Degelia	0.320	0.005**
Mollisina	0.319	0.020*
Dominikia	0.317	0.005**
Scleropezicula	0.314	0.030*
Operculomyces	0.313	0.025*
Athelopsis	0.309	0.045*
Varicellaria	0.299	0.030*
Apodus	0.292	0.035*
Aspergillus	0.263	0.025*
Fuscidea	0.263	0.015*
Rhizophagus	0.263	0.035*
Ramariopsis	0.262	0.025*
Furcasterigmium	0.258	0.035*
Hohenbuehelia	0.255	0.040*
Protomerulius	0.254	0.050*
Tubaria	0.252	0.045*
Peristomialis	0.249	0.045*
Cryptodiscus	0.249	0.040*
Clonostachys	0.223	0.025*

*Note:* Asterisks denote significant *p*‐values.
